# The Importance of Being a ‘Lark’ in Post-Menopausal Women with Obesity: A Ploy to Prevent Type 2 Diabetes Mellitus?

**DOI:** 10.3390/nu13113762

**Published:** 2021-10-25

**Authors:** Luigi Barrea, Claudia Vetrani, Barbara Altieri, Ludovica Verde, Silvia Savastano, Annamaria Colao, Giovanna Muscogiuri

**Affiliations:** 1 Dipartimento di Scienze Umanistiche, Università Telematica Pegaso, Via Porzio, Centro Direzionale, isola F2, 80143 Naples, Italy; luigi.barrea@unina.it; 2 Centro Italiano per la Cura e Il Benessere del Paziente con Obesità (C.I.B.O.), University Federico II, Via Sergio Pansini 5, 80131 Naples, Italy; sisavast@unina.it (S.S.); colao@unina.it (A.C.); 3 Endocrinology Unit, Department of Clinical Medicine and Surgery, University Federico II, 80131 Naples, Italy; c.vetrani@libero.it (C.V.); ludoverde96@gmail.com (L.V.); 4 Division of Endocrinology and Diabetes, Department of Internal Medicine I, University Hospital, University of Würzburg, 97080 Würzburg, Germany; Altieri_B@ukw.de; 5 Cattedra Unesco “Educazione Alla Salute e Allo Sviluppo Sostenibile”, University Federico II, 80131 Naples, Italy

**Keywords:** chronotype, circadian rhythms, menopause, obesity, type 2 diabetes, cardiovascular diseases

## Abstract

Chronotype is defined as the behavioral manifestation of circadian rhythms related to the external light–dark cycle. Evening chronotype has been associated with an increased risk of developing cardiometabolic diseases in obesity. Menopause is a lifestage associated with an increased risk of developing cardiometabolic diseases and a change in circadian rhythmicity compared to pre-menopause. However, the prevalence of chronotype categories in menopause and their role in determining menopause-related cardiometabolic risk, mostly in obesity, have not been investigated. Thus, we aimed to investigate the prevalence of chronotype categories in post-menopausal women with obesity and their role in menopause-related cardiometabolic risk. In this cross-sectional study we enrolled 49 pre-menopausal and 74 post-menopausal women with obesity. Anthropometric parameters, lifestyle habits, adherence to the Mediterranean Diet (MD), sleep quality, chronotype and the presence of type 2 diabetes mellitus (T2DM) and cardiovascular diseases (CVD) were studied. No significance differences were detected in terms of lifestyle and adherence to the MD between pre- and post-menopausal women. Chronotype was classified as morning in 66 (53.6%), evening in 20 (16.3%) and intermediate in 37 (30.1%) women. In addition, pre-menopausal women with obesity showed a significantly higher chance to have an intermediate chronotype (OR = 2.21, 95% CI 1.28–3.83; *p* = 0.004), whereas post-menopausal women with obesity showed a trend to have a higher morning chronotype (OR = 1.42, 95% CI 0.98–2.06; *p* = 0.051), although this did not reach statistical significance. No significant differences were detected in terms of prevalence of evening chronotype between the two groups. However, the evening chronotype had a significantly higher risk to have T2DM compared to the morning (OR = 17.29, 95% CI 2.40–124.27; *p* = 0.005) and intermediate chronotypes (OR = 30.86, 95% CI 2.05–464.32; *p* = 0.013) in both pre- and post-menopausal women with obesity. In conclusion, the intermediate chronotype was significantly more prevalent in pre-menopausal women with obesity compared to post-menopausal women. Evening chronotype was associated to T2DM in both pre- and post-menopause. These results support the importance of including the assessment of chronotype in the management of women with obesity in post-menopause.

## 1. Introduction

The behavioral manifestation of the internal circadian clock system related to the external light–dark cycle is commonly referred to as the chronotype [1,2]. There are three general categories of chronotypes based on variants of the circadian behavioral phenotype: morning, evening, and intermediate chronotypes [3]. The morning chronotype (defined as “larks”) tends to wake up early and prefers activities earlier in the day, while the evening chronotype (defined as “owls”) generally wakes up later and prefers to perform major activity in the late afternoon or evening. Intermediate chronotype is an intermediate position between the morning and evening chronotypes [3]. The evening chronotype encounters health problems such as psychological disorders and gastrointestinal diseases more often and has a higher mortality rate than the morning chronotype [4,5]. In addition, the evening chronotype has been reported to be more prone to develop metabolic diseases such as type 2 diabetes (T2DM) and metabolic syndrome than the morning chronotype [6].

In agreement with these findings, data coming from the OPERA (Obesity, Programs of nutrition, Education, Research and Assessment of the best treatment) Prevention Project pointed out that individuals with the evening chronotype were, when compared to intermediate and morning chronotypes, more prone to follow an unhealthy lifestyle, performing less regular activity and being more frequent smokers [7,8]. In addition, they showed the lowest adherence to the Mediterranean Diet (MD) compared to the morning and intermediate chronotypes, and the lower their chronotype score, the higher the Body Mass Index (BMI) values in all populations, thus suggesting that the highest chronotype score was more commonly detected in subjects with obesity [9]. Beyond obesity, menopause has been identified as a life stage in women associated with an increased risk of developing cardiometabolic diseases [10].

Menopause is a physiological event in the life of a woman characterized by the permanent cessation of ovarian follicular activity which causes an abrupt drop in estrogen levels, resulting in the classic signs and symptoms of menopause [11]. Moreover, menopause is a risk factor for cardiovascular disease (CVD) because the drop in estrogen impairs cardiovascular function and metabolism [12]. Indeed, menopause predisposes women to the development of many traditional CVD risk factors. These include changes in body fat distribution from a gynoid to an android pattern, decreased glucose tolerance, abnormal plasma lipids, increased blood pressure, increased sympathetic tone, endothelial dysfunction, and vascular inflammation [12].

In addition, post-menopausal women show a loss of circadian robustness and an increase in sleep disturbance [13,14], a process that parallels the redistribution of adipose tissue [13]. Indeed, menopause alters the profile of clock gene expression in adipose tissue [15], possibly related to the redistribution of body fat after menopause, which in turn is characterized by an increase in visceral fat. Estradiol can act through both organizational and activating mechanisms [16]. The organizational mechanism is related to the effects of prenatal exposure to sex hormones that are able to determine that the response to gonadal hormones in adulthood [16]. Thus, it can be hypothesized that when genes that regulate circadian functions lose some of their precise orchestration with aging, it leads to disturbed homeostasis. 

Given the role of chronotype in determining the risk of developing cardiometabolic diseases, and given the fact that menopause is a lifestage associated with an increased risk of developing cardiometabolic diseases compared to pre-menopause, we aimed to investigate the prevalence of chronotype categories in post-menopausal compared to pre-menopausal women with obesity and the role of chronotype in determining menopause-related cardiometabolic diseases. 

## 2. Materials and Methods

For this cross-sectional study, we used data collected during “the OPERA (Obesity, Programs of nutrition, Education, Research and Assessment of the best treatment) Prevention Project”, held in Naples from October 11 to 13, 2019 [7]. The methods and procedure of data collection during the event are described in detail elsewhere [7]. In the present study, we analyzed data from 123 women (BMI 31.7 ± 6.3; age 51.1 ± 16.0 years). Of these, 49 (34.9 ± 10.6 years) were pre-menopausal and 74 (61.8 ± 7.6 years) were post-menopausal. Middle-aged women with normal liver, cardiopulmonary, and renal functions were eligible as participants for the study. We also excluded females who were taking medications for liver, kidney, and cardiopulmonary diseases. All subjects gave written informed consent to the study, which was conducted according to the Helsinki Declaration for Human Studies.

### 2.1. Sample Size Justification and Power

In the absence of similar clinical studies available in the literature, the calculation of the sample size was performed a priori by considering the effect size 0.8 with type I error of 0.05 and a power of 90%. The number of subjects to be enrolled was found to be 34 per group. During the campaign, 123 women were deemed eligible for the study. Since this sample not only met at least the necessary number of subjects, but also further supported the results, we decided to include all subjects in the statistical analysis. The calculation of the sample size was performed using G Power Software.

### 2.2. Data Collection

Anthropometric parameters, demographic information, and chronotype assessment were collected. We defined “current smoker” if subjects reported smoking at least one cigarette per day and “nonsmoker” if subjects reported not smoking. Participants who habitually engaged in at least 30 min/day of aerobic exercise (YES/NO) were defined as physically active, as we have reported in detail in previous studies [17,18,19]. Adherence to the MD was assessed using the 14-item “PREvencion con DIeta MEDiterranea Evaluation Questionnaire” (PREDIMED), as previously reported [20]. Enrolled women were asked whether they were affected by T2DM and/or CVD (myocardial infarction, stroke, hypertension).

### 2.3. Menopause Diagnosis

Menopause was diagnosed in healthy women over 45 years of age who: 1) had been amenorrhoeic for at least 12 months in the absence of treatment with hormonal contraceptives, or 2) were hysterectomised women with menopausal symptoms (e.g., vasomotor, musculoskeletal and urogenital symptoms, mood changes, sexual problems) [14].

### 2.4. Anthropometric Parameters

Women were wearing only light clothing and no shoes when anthropometric parameters were assessed, as previously reported [21,22,23]. The formula of BMI was the following: weight (kg)/height (m^2^). A wall-mounted stadiometer was used to assess height. A calibrated scale was used to assess body weight. Waist circumference (WC) was measured to the nearest 0.1 cm using a non-stretchable tape. Obesity I grade was defined when BMI was between 30 and 34.9 kg/m^2^, obesity II grade when BMI was between 35 and 39.9 kg/m^2^, and obesity III grade when BMI was equal to or greater than 40.0 kg/m^2^.

### 2.5. Assessment of Chronotype 

Morningness–eveningness was measured with the Horne–Ostberg Morningness–Eveningness Questionnaire (MEQ) [24]. The MEQ consisted of 19 questions about preferred sleep time and daily performance. Scores ranged from 16 to 86, and based on their scores, women were categorized as morning (59–86), neither (42–58), or evening (16–41) types.

### 2.6. Assessment of Sleep

Overall sleep quality was assessed using the Pittsburgh Sleep Quality Index (PSQI) [25]. Poor sleepers were defined as women with a PSQI score of 5. Good sleepers were defined as women with a PSQI score of <5.

### 2.7. Statistical Analysis

Data distribution was evaluated using the Shapiro–Wilk test. A two-sided t-test or Mann–Whitney test was used to compare continuous variables, which are shown as the mean ± standard deviation [14]. The Chi-square (χ^2^) test or Fisher’s exact test was used to determine the differences in the frequency distribution of dichotomous variables, which are shown as numbers and percentages. Multivariate logistic regression analyses were performed to assess associations between chronotype and the presence of T2DM and CVD after adjustment for menopause, BMI, smoke, dyslipidemia, and sleep quality (model 1), and menopause, BMI, smoke, dyslipidemia, sleep quality, and PREDIMED score (model 2). Data were analysed using SPSS Software (PASW Version 21.0, SPSS Inc., Chicago, IL, USA) and GraphPad Prism (version 5.0, La Jolla, CA, USA). A *p* value < 0.05 was considered statistically significant.

## 3. Results

One hundred and twenty-three females (51.1 ± 16.0 years) were included. Fourty-nine were pre-menopausal (34.9 ± 10.6 years) and 74 were post-menopausal (34.9 ± 10.6 years). Table 1 lists the clinical characteristics, lifestyle habits and anthropometric measurements of the cohort of patients. As shown in Table 1, the mean BMI was 31.7 ± 6.3 kg/m^2^. Specifically, 15 (12.2%) women were normal weight, 33 (26.8%) were overweight, and 41 (33.3%), 22 (17.9%) and 12 (9.8%) women had grade I, II and III obesity, respectively. Mean WC of enrolled females was 99.7 ± 15.5 cm while mean waist to hip ratio (WHR) was 0.9 ± 0.1. Most of the women had a morning chronotype type (66; 53.6%) and an average adherence to the MD (PREDIMED score: 7 ± 2). Sixty-six (53.7%) were sedentary and 65 (52.8%) poor sleepers. Twenty-five (20.3%) were smokers. Finally, in our cohort of females, 31 (25.2%) had dyslipidemia, 13 (10.6%) had T2DM and 34 (27.6%) had CVD.

As expected, age significantly differed between pre- and post-menopausal women (34.9 ± 10.6 and 61.8 ± 7.6 years; *p* < 0.0001 respectively). While BMI did not differ between the two groups, the percentage of normal-weight women was significantly higher in the group of pre-menopausal women than in the group of post-menopausal women (*p* = 0.022). In addition, the latter also had a statistically significant higher WHR (*p* = 0.01). Obesity was present in most of the enrolled females in both groups: specifically, in the pre-menopausal group, grade I obesity was present in 15 (30.6%), grade II obesity in 12 (24.5%), and grade III obesity in 5 (10.2%) females. In the post-menopausal group, grade I obesity was found in 26 (35.1%), grade II obesity in 10 (13.5%), and grade III obesity in 7 (9.5%) women. The mean PREDIMED score was similar in both groups. Twenty-four (49.0%) in the pre-menopausal group and 41 (55.4%) in post-menopausal group were poor sleepers, with an average sleep duration of 6.5 ± 1.5 and 5.9 ± 1.6 h, respectively. Eight (16.3%) pre-menopausal women and 17 (23%) post-menopausal women had smoking habits, while 21 (42.9%) of the pre-menopausal group and 36 (48.6%) of the post-menopausal group performed moderate physical activity. Dyslipidemia was significantly more common in post-menopausal women compared to pre-menopausal women (28; 37.8% vs. 3; 6.1%; *p* < 0.001) as well as CVD (8; 16.3% vs. 8; 16.3%; *p* = 0.022). No significant differences were detected in terms of prevalence of T2DM (3; 6.1%; vs. 10; 13.5%; *p* = 0.19) between the two groups.

### 3.1. Women’s Characteristics According to Chronotype

The chronotype categories’ prevalence significantly varied between pre-menopausal and post-menopausal women. In the pre-menopausal group 21 (42.9%) women had a morning chronotype, 22 (44.9%) had a medium chronotype and 6 (12.2%) had an evening chronotype, while in the post-menopausal group 45 (60.8%) women had a morning chronotype, 15 (20.3%) had a medium chronotype and 14 (18.9%) had an evening chronotype. Particularly, pre-menopausal women were more frequently an intermediate chronotype (OR = 2.21, 95% CI 1.28–3.83; *p* = 0.004), whereas post-menopausal women showed a trend to more frequently have a morning chronotype (OR = 1.42, 95% CI 0,98–2.06; *p* = 0.051), although it did not reach statistical significance. It would appear that post-menopausal women have a slightly lower risk of having an evening chronotype (OR = 0.60; 95% CI 0.23-1.63; *p* = 0.45), although this also did not reach statistical significance.

Chronotypes were significantly different between pre-menopausal and post-menopausal women. Statistical analysis was performed by Chi-square test.

### 3.2. Association of Chronotype with T2DM and CVD

The association between chronotype and the presence of T2DM and CVD is reported in Table 2. After adjusting the analysis for menopause, BMI, smoking, dyslipidemia, and sleep quality (Model 1), the evening chronotype had a significantly higher risk to have T2DM in both pre- and post-menopausal women compared to the morning (OR = 17.29, 95% CI 2.40–124.47; *p* = 0.005) and intermediate chronotypes (OR = 30.86, 95% CI 2.05–464.32; *p* = 0.013). These associations remained significant after adjustment for adherence to MD (Model 2; Table 2). No significant differences were observed between morning and intermediate chronotypes in either model.

## 4. Discussion

The main purpose of this study was to investigate the prevalence of chronotype categories in post-menopausal women with obesity compared with pre-menopausal women with obesity. As novel findings, our results showed that the prevalence of chronotype categories varied significantly between pre-menopausal and post-menopausal women. Specifically, pre-menopausal women showed a significantly higher chance of having an intermediate chronotype, whereas post-menopausal women showed a tendency to have a morning chronotype, although it did not reach statistical significance. These results are consistent with those of previous studies. Specifically, Gomez Santos et al. reported that post-menopausal women tended to be more morning type in behavior and physiology compared with pre-menopausal women [26,27]. In fact, post-menopausal women woke up earlier and ate breakfast earlier than pre-menopausal women, and increasing age is related to a higher morningness score [26,27]. On the other hand, menopause is characterized by a change in lifestyle that generally occurs with age. It is likely that pre-menopausal women tend to have a less pronounced morning chronotype, partly due to a more hectic lifestyle and more commitments during the day; it is known that chronotype partially depends on environmental factors [28,29,30]. In addition, chronotype is related to age: children are generally morning chronotypes [31]. As they grow older, they become more and more evening types, until a peak around the age of 20, after which they become progressively more morning types again. In addition, a gender difference in the chronotype has been reported [32]; the general tendency for women to have puberty earlier than men is also reflected in the chronotype, where young women become evening types earlier than young men. This gender difference disappears around the age of 50, which coincides with the average age of menopause [31]. This could be due to a combination of less regular social and light schedules or a less robust circadian system in later life [33,34]. Since post-menopause is accompanied by hormonal changes compared to pre-menopause, it could be hypothesized that hormonal changes could have a role in the change of chronotype categories over the years. [35]. Indeed, the concentration and timing of release of many hormones are age-dependent [36]. In addition, estrogen also has an antidepressant effect. With less estrogen, women may experience higher body temperatures, lower quality sleep and poorer mood [37]. The sleep–wake cycle also changes with increasing age and loses its consistency. Post-menopausal women begin to feel tired earlier and wake up earlier in the morning, leading to less sleep overall [26]. In agreement with these data, we found that post-menopausal women slept 10% fewer hours than pre-menopausal women [26]. Studies of sleep quality during the menopausal transition and post-menopause revealed that lower estradiol levels were associated with night-time awakening [38]. Findings from the Study of Women’s Health Across the Nation [39] indicated that lower estradiol levels and higher FSH were associated with difficulty falling asleep and remaining asleep [40]. Furthermore, these results were confirmed in later studies of polysomnographic sleep in a subgroup of SWAN participants [39]. In fact, in this study increased beta-EEG power in NREM sleep was shown in women transitioning to post-menopause, suggesting that the menopausal transition is associated with physiological overexcitation during sleep independent of self-reported hot flashes [39]. A relationship between sleep changes and cortisol with ageing has been reported [41,42]. Healthy older adults have elevated cortisol levels at the circadian nadir and have higher basal cortisol levels than younger adults [41]. The circadian amplitude of many variables, including cortisol [41] and temperature [42], decreases with age in both women and men. In adults, cortisol levels are lower in pre-menopausal females than in males of a similar age [41]. After menopause, there are no distinguishable gender differences in total plasma cortisol levels [41]. Thus, these data could explain the tendency towards a morning chronotype in post-menopausal women. Cortisol is the stress hormone [43] and in addition, gender differences in stress response have been hypothesized [44]. Older women, when subjected to mild stress, show greater effects in sleep parameters than men of the same age, specifically manifesting an increase in sleep latency, a decrease in time in bed, a decrease in total sleep time and an increase in total wake time [44]. In fact, recent work has shown that the circadian pacemaker in the central nervous system is anatomically and functionally linked to the paraventricular neurons that control the release of CRH and ACTH [45], which are known to be activated by stress. In light of this evidence, stress reactivity could be an important factor contributing to age-related sleep quality and circadian changes. In addition, these change in cortisol secretion could contribute to menopause related changes in body composition. Indeed, it is known that menopause is characterized by an increase in visceral adipose tissue that in turn increases the risk of cardiometabolic disease [46]. Similarly, we found that post-menopausal women had an increased prevalence of dyslipidemia and CVD.

Secondly, our data confirm that the evening chronotype in both pre- and post-menopause represents a risk factor for the development of T2DM. This result is in line with other evidence from the literature [47,48,49,50,51,52]. In this regard, data from the Sleep Extension Study suggest that individuals with the evening chronotype tend to eat later and eat fewer and larger meals, which are known risk factors for glucose metabolism disorders [48]. Our findings are consistent with a previous study using data from the National FINRISK Study 2007, a representative sample of the population aged 25-74 years living in five regions of Finland [49]. Evening chronotypes had a 2.5-fold OR for T2DM compared with morning types, and this association was independent of sleep duration and sleep sufficiency [49]. It has been reported that in humans, glucose tolerance tends to decrease from morning to evening due to a combination of decreased glucose utilization, decreased insulin sensitivity, and inappropriately low insulin secretion [52]. The evening chronotype usually eats a rich dinner, thus matching the glucose metabolism in its worsening daily performance. The effects of shifting circadian rhythms were studied in 10 adults (5 women) who underwent a 10-day laboratory protocol in which subjects ate and slept during all phases of the circadian cycle, which was accomplished by scheduling a recurring 28-h “day” [51]. 

Our study has several strengths, of which the following are worthy of note. First, to our knowledge, the current study is the first to examine prevalence of chronotype categories in pre-menopausal or post-menopausal women with obesity and their association with cardiometabolic diseases. Identifying post-menopausal women with an evening chronotype is of paramount importance as they are at increased risk of developing T2DM and therefore need to be closely monitored. Furthermore, an intervention aimed at bringing the evening chronotype setting back into line with circadian time could be an effective attempt to prevent the onset of the T2DM. Second, we used a widely used, validated questionnaire to collect data on chronotypes [9,24]. Interestingly, the MEQ questionnaire allows for feedback to participants immediately after completion of the survey. Finally, we included well-selected women with strict inclusion/exclusion criteria and both groups were well characterized. The limitation of the study is mainly its cross-sectional experimental design which, while showing an association of the evening chronotype with an increased risk of T2DM in both pre- and post-menopausal women, fails to provide any explanation of the causality of this association. Furthermore, this was a single-centre study, so participants were from the same geographical area and probably had similar food availability and eating habits, which may limit the transfer of these data to other populations.

## 5. Conclusions

Pre- and post-menopausal women with obesity have a different chronotypes, more frequently an intermediate type in pre-menopausal women and more frequently a morning type in post-menopausal women. Post-menopausal women who escape this physiological chronotype alignment towards a more morning type and become evening chronotypes may have an increased risk of developing T2DM. In the context of both pre- and post-menopausal women, the evening chronotype is confirmed as a risk factor for T2DM, thus contributing further to that already provided by obesity and menopause. These results highlight the importance of including chronotype assessment as an additional tool in the assessment and management of post-menopausal women with obesity.

## Figures and Tables

**Table 1:** Clinical characteristics, lifestyle habits, anthropometric measurements, adherence to the MD, sleep quality and chronotype categories in all cohorts and according to pre/post-menopause.

Parameters	All Women (*n* = 123)	Pre-Menopausal Women (*n* = 49)	Post-Menopausal Women (*n* = 74)	*p* Value, χ^2^
Age (years)	51.1 ± 16.0	34.9 ± 10.6	61.8 ± 7.6	**<0.0001**
BMI (kg/m^2^)	31.7 ± 6.3	31.9 ± 7.2	31.5 ± 5.6	0.55
BMI categories:				
Normal weight	15 (12.2%)	10 (20.4%)	5 (6.8%)	**0.022, 11.46**
Overweight	33 (26.8%)	7 (14.3%)	26 (35.1%)	
Obesity I	41 (33.3%)	15 (30.6%)	26 (35.1%)	
Obesity II	22 (17.9%)	12 (24.5%)	10 (13.5%)	
Obesity III	12 (9.8%)	5 (10.2%)	7 (9.5%)	
Waist circumference (cm)	99.7 ± 15.5	97.1 ± 18.5	101.5 ± 12.9	0.36
WHR	0.9 ± 0.1	0.8 ± 0.1	0.9 ± 0.1	**0.01**
Chronotype:				
Morning	66 (53.6%)	21 (42.9%)	45 (60.8%)	
Intermediate	37 (30.1%)	22 (44.9%)	15 (20.3%)	**0.014, 8.52**
Evening	20 (16.3%)	6 (12.2%)	14 (18.9%)	
Hours of sleeping	6.1 ± 1.5	6.5 ± 1.5	5.9 ± 1.6	0.08
Pittsburgh score-categories:				
Good sleepers	58 (47.2%)	25 (51.1%)	33 (44.6%)	0.48, 0.49
Poor sleepers	65 (52.8%)	24 (49.0%)	41 (55.4%)	
PREDIMED score	7 ± 2	8 (3–11)	8 (2–13)	0.76
PREDIMED categories				
Low adherence	16 (13.0%)	5 (10.2%)	11 (14.9%)	0.18, 3.45
Average adherence	81 (65.8%)	37 (75.5%)	44 (59.5%)	
Highest adherence	26 (21.2%)	7 (14.3%)	19 (25.7%)	
Physical activity:				
Sedentary	66 (53.7%)	28 (57.1%)	38 (51.4%)	0.53, 0.39
Moderate	57 (46.3%)	21 (42.9%)	36 (48.6%)	
Smoke:				
Non smokers	98 (79.7%)	41 (83.7%)	57 (77.0%)	0.37, 0.80
Current smokers	25 (20.3%)	8 (16.3%)	17 (23.0%)	
Dyslipidemia:				
No dyslipidemia	92 (74.8%)	46 (93.9%)	46 (62.2%)	**<0.001, 15.7**
Dyslipidemia	31 (25.2%)	3 (6.1%)	28 (37.8%)	
Type 2 diabetes mellitus:				
No T2DM	110 (89.4%)	46 (93.9%)	64 (85.5%)	0.19, 1.70
T2DM	13 (10.6%)	3 (6.1%)	10 (13.5%)	
Cardiovascular disease:				
No CVD	89 (72.4%)	41 (83.7%)	48 (64.9%)	**0.022, 5.21**
CVD	34 (27.6%)	8 (16.3%)	26 (35.1%)	

Results were expressed as number (%) or mean ± SD. A *p* value in bold type denotes a significant difference (*p* < 0.05). BMI, body mass index; WHR, waist to hip ratio; PREDIMED, PREvención con DIeta MEDiterránea; T2DM, type 2 diabetes mellitus; CVD, cardiovascular disease.

**Table 2:** Association of chronotype categories with T2DM and CVD.

**Model 1 (without PREDIMED as a Covariate)**			
**Morning chronotype vs. intermediate chronotype**			
	*p*	OR	95% CI
T2DM	0.24	4.50	0.36–56.58
CVD	0.35	1.74	0.55–5.51
**Evening chronotype vs. morning chronotype**			
	*p*	OR	95% CI
T2DM	0.005	17.29	2.40–124.47
CVD	0.17	2.65	0.67–10.53
**Evening chronotype vs.intermediate chronotype**			
	*p*	OR	95% CI
T2DM	0.013	30.86	2.05–464.32
CVD	0.42	1.92	0.40–9.28
**Model 1 (with PREDIMED as a covariate)**			
**Morning chronotype vs. intermediate chronotype**			
	*p*	OR	95% CI
T2DM	0.25	4.24	0.36–49.79
CVD	0.63	1.34	0.41–4.39
**Evening chronotype vs. morning chronotype**			
	*p*	OR	95% CI
T2DM	0.026	13.67	1.36–137.34
CVD	0.11	3.86	0.74–20.34
**Evening chronotype vs. intermediate chronotype**			
	*p*	OR	95% CI
T2DM	0.028	20.23	1.39–293.99
CVD	0.25	3.07	0.45–21.13

Logistic regression analysis to evaluate the associations between chronotypes and the presence of type 2 diabetes mellitus and cardiovascular disease after considering menopause, BMI, smoking, dyslipidemia, and sleep quality as covariates (model 1) or after considering menopause, BMI, smoke, dyslipidemia, sleep quality, and PREDIMED score as covariates (model 2). Significant p values are reported in bold. BMI, body mass index; T2DM, type 2 diabetes mellitus; PREDIMED, PREvención con DIeta MEDiterránea; CVD, cardiovascular disease.

## Data Availability

Results attained in this study are included in the manuscript. Individual data are not publicly available due to ethical restrictions.
